# Is bilingualism losing its advantage? A bibliometric approach

**DOI:** 10.1371/journal.pone.0176151

**Published:** 2017-04-20

**Authors:** Victor A. Sanchez-Azanza, Raúl López-Penadés, Lucía Buil-Legaz, Eva Aguilar-Mediavilla, Daniel Adrover-Roig

**Affiliations:** Department of Applied Pedagogy and Educational Psychology, Universitat de les Illes Balears, Illes Balears (Spain); Western University, CANADA

## Abstract

This study uses several bibliometric indices to explore the temporal course of publication trends regarding the bilingual advantage in executive control over a ten-year window. These indices include the number of published papers, numbers of citations, and the journal impact factor. According to the information available in their abstracts, studies were classified into one of four categories: supporting, ambiguous towards, not mentioning, or challenging the bilingual advantage. Results show that the number of papers challenging the bilingual advantage increased notably in 2014 and 2015. Both the average impact factor and the accumulated citations as of June 2016 were equivalent between categories. However, of the studies published in 2014, those that challenge the bilingual advantage accumulated more citations in June 2016 than those supporting it. Our findings offer evidence-based bibliometric information about the current state of the literature and suggest a change in publication trends regarding the literature on the bilingual advantage.

## Introduction

In recent years, the behavioral performance of monolingual and bilingual participants during tasks related to executive control has become a theme of debate [1] and has emerged as a controversial topic in cognitive science. In general, the results accumulated over the past years support the idea that bilinguals show enhanced cognitive control capacity when compared to their monolingual peers. This phenomenon has been labelled the ‘bilingual advantage’ (BA), and it has been reported in different population groups, such as children [2], young adults [3], and older adults [4]. In this regard, the BA has been assumed to stem from the proficient ability to switch between two or more languages, which involves both the inhibition of one language and the subsequent activation of the target language during oral production [5]. This continuous use of language alternation may extend to other cognitive processes beyond language [6]. In particular, bilinguals have been shown to outperform monolinguals in tasks related to specific aspects of cognitive control, such as the inhibition of irrelevant information [7], the switch towards new relevant information [8], and the updating of information in working memory [9].

Early studies on the cognitive consequences of bilingualism showed a different pattern of results. Through the first 60 years of the 20th century, most of the research on the relationship between bilingualism and cognition focused on the influence of bilingualism on the intelligence of children. In this regard, findings typically showed higher or similar scores in measures of intellectual functioning for monolingual children as compared to their bilingual counterparts (for a review, see [10]). However, as some authors have pointed out, these studies contained several methodological flaws, such as frequent inappropriate matching of the groups [11,12]. Since Peal and Lambert [13] challenged the then pre-existing notions by showing that bilingual children performed better in verbal and nonverbal measures of intelligence, the literature in favor of the BA hypothesis has been abundant. In particular, most research has focused on the relationship between bilingualism and cognitive control in order to disentangle its key aspects by examining diverse features, such as language proficiency [14], age of second language acquisition [15], brain network organization [16], age [4], or individual differences in the use of language by bilinguals [17], among others.

Although the majority of published studies have reported results that support the BA hypothesis, some studies have shown a different pattern of results, reporting null, mixed, or even reversed effects between bilinguals and monolinguals in tasks related to cognitive control [18,19]. Despite the fact that there are studies that refute the assumptions of the BA hypothesis, the existence of a publication bias in favor of positive results has been recently suggested analyzing studies published until early 2014 [20]. However, the number of studies reporting results that challenge the BA hypothesis may have increased over the last few years [21–25], especially following the influential publication of Paap and Greenberg [1] in 2013. These authors tested the BA hypothesis in a large sample of bilinguals using a set of different tasks designed to assess the three cognitive control mechanisms that are purportedly enhanced in bilinguals (*i*.*e*., inhibition, switching, and updating of information). Their results revealed null differences when bilinguals were compared to monolinguals, while a better performance trend was found for the monolingual group under some conditions of the different tasks. Moreover, the authors exposed two interesting aspects of the methodology used in studies pertaining to the BA literature: the lack of success in replicating some studies showing better performance in bilinguals, which may be due to task-dependent effects evidenced by a low convergent validity with other tasks assessing the same components of cognitive control (mainly inhibitory control; see also [26] for a more exhaustive discussion regarding this issue); and not strictly controlling demographic variables, such as socioeconomic status [27], which may be at the base of some group differences found in the BA literature.

In light of the above mentioned controversy, the purpose of the present study is to objectively test the impression that a change in publication trends is taking place in the literature regarding the BA. Accordingly, our aim was not to take part in this on-going debate, but to provide an objective bibliometric analysis on the relationship between bilingualism and cognitive control in recent scientific literature. Bibliometrics is a quantitative statistical technique used to measure levels of production and dissemination of knowledge, as well as a useful tool for tracking the progress of a scientific area [28]. In the present study, we conduct an in-depth bibliometric analysis of the literature on the BA hypothesis before and after 2014. To do so, we carry out an online search for studies concerning the influence of bilingualism on cognitive control and classify them into four categories according to their behavioral results: the SBA category (supports the BA hypothesis), the ABA category (ambiguous regarding the BA hypothesis), the NMA category (does not mention its positioning regarding the BA hypothesis), and the CBA category (challenges the BA hypothesis). Then, we examine the temporal evolution of each publication category on several bibliometric indices, with particular interest in the SBA and CBA categories. Following de Bruin and Della Sala [20], we expect studies challenging the BA hypothesis to attain similar or even higher bibliometric scores from 2014 onwards, compared to published papers that support the BA. Such a finding would provide evidence that there has been a change in publication trends regarding the BA hypothesis.

## Method

The search was conducted in January 2016, using the Scopus database to find papers investigating the relationship between bilingualism and cognitive control over the previous 10 years. The results of this search included papers published between 2005 and 2015, and the query included the following terms in their abstracts, titles, or keywords: bilingualism, cognitive, control, executive, interference, and advantage (the actual query used was: “TITLE-ABS-KEY(bilingual*) AND TITLE-ABS-KEY(cognitive) OR TITLE-ABS-KEY(control) OR TITLE-ABS-KEY(executive) OR TITLE-ABS-KEY(interference) AND TITLE-ABS-KEY(advantage) AND PUBYEAR > 2004 AND PUBYEAR < 2016 AND DOCTYPE(ar))”). As a result, 189 papers were retrieved.

### Paper categories, inclusion and exclusion criteria, and bibliometric indices

As our principal concern was to provide an overview of the current publication trends regarding the BA in healthy samples, we omitted papers that included the following features in absence of healthy groups comparisons: neurological or psychiatric disorders (aphasia, MCI/AD, SLI), script perspective (acquisition of an artificial logographic script), and bimodal bilingualism. Papers without a cognitive perspective (teacher collaboration in bilingual contexts) and reviews were also excluded from the analyses.

As a result, a total of 139 papers were considered after applying all exclusion criteria. Following de Bruin et al. [20], papers were categorized based only on the behavioral information provided in their respective abstracts. When we use the term ‘behavioral information’ we refer to any mention of a result (*e*.*g*., reduced switch costs in bilinguals compared to monolinguals) or a conclusion in absence of specific results (*e*.*g*., bilinguals outperforming monolinguals) involving comparisons between bilingual and monolingual groups on direct or derived behavioral measures captured by task conditions. In this vein, we counted each supporting (result of tasks showing an advantage for the bilingual group) or challenging (result of tasks showing an advantage for the monolingual group, or null results) behavioral information reported in the abstracts, and computed a percentage for every study. Then, we used this percentage to classify the abstract into the category it belonged (see Table 1). We did not take into account the interpretations drawn by the authors in order to classify any of the studies. Rather, we did only focus on the behavioral information provided in their respective abstracts.

**Table 1:** Study categorization criteria, examples, and descriptive statistics for each paper category regarding the percentage of behavioral information supporting the BA.

Category	Percentages		Example 1			Example 2		
	%Sup	Mean (SD)	nSup	nCha	%Sup	nSup	nCha	%Sup
SBA	≥ 80	98.14 (5.88)	3	0	100	5	1	83
ABA	21–79	50.9 (12.91)	2	2	50	2	4	33
NMA	-	-	-	-	-	-	-	-
CBA	≤ 20	1.96 (5.88)	0	3	0	1	6	14

SBA: supports the bilingual advantage; ABA: ambiguous about the bilingual advantage; NMA: does not mention the bilingual advantage; CBA: challenges the bilingual advantage; %Sup: percentage of behavioral information supporting the BA in the abstract; nSup: number of behavioral results supporting the BA in the abstract; nCha: number of behavioral results challenging the BA in the abstract (reversed or null results); SD: standard deviation.

Each paper was classified by two independent raters into one of four exclusive categories. Although there was a strong agreement between the two raters, *κ* = 0.824 (95% CI, .747 to .9), *p* < .0001, all discrepancies (18 cases) were resolved by discussion. The first category included all the papers that supported the bilingual advantage (SBA) with at least 80% of the behavioral information reported in the abstract pointing to the outperformance of bilinguals over monolinguals. The second category contained papers that were ambiguous regarding the bilingual advantage (ABA). In these studies, some behavioral results showed advantages for the bilingual group (in a range of 50% to 79%), while some favored their monolingual counterparts or referenced null results (in a range of 21% to 50%), or vice versa. The third category was made up of studies in which the bilingual advantage was not mentioned (NMA). Abstracts that did not mention group comparisons between monolinguals and bilinguals, and focused only on comparisons within bilingual participants (*e*.*g*., switchers vs. non-switchers) were also included in the NMA category, regardless of their position on the BA. The fourth category consisted of studies challenging the bilingual advantage (CBA), in which at least an 80% of the behavioral information reported in the abstract showed no difference between monolingual and bilingual groups, or where advantages for monolinguals were reported. Studies with abstracts that did not find a behavioral advantage for bilinguals and seemed to support the BA hypothesis based merely on neuroimaging or event-related potentials data were also placed in the CBA category. Following this criterion, 5 studies (17% of the CBA category) were classified as CBA, although the conclusions in their abstracts appeared to support the BA. Descriptive data for each category can be consulted in Table 2.

**Table 2:** Number of studies, percentages, accumulated citations, mean accumulated citations and mean impact factor, classified by paper category, and publication time period.

		SBA	ABA	NMA	CBA
Total					
N		43	42	25	29
% of papers		30.9	30.2	18	20.9
Acc. citations (June 2016)		715	1696	349	562
Mean acc. citations (June 2016)		16.6	40.4	13.9	19.4
Mean JIF		2.8	2.4	2.9	2.5
Time period					
N	2005–2013	26	27	16	9
	2014–2015	17	15	9	20
% of papers	2005–2013	60.5	64.3	64	31
	2014–2015	39.5	35.7	36	69
Acc. citations	2005–2013	655	1638	326	382
	2014—June 2016	60	58	23	180
Mean acc. citations	2005–2013	25.2	60.7	20.4	42.4
	2014—June 2016	3.5	3.9	2.6	9
Mean JIF	2005–2013	2.9	2.7	3.1	3.1
	2014–2015	2.5	1.9	2.7	2.3

Acc.: accumulated; SBA: supports the bilingual advantage; ABA: ambiguous about the bilingual advantage; NMA: does not mention the bilingual advantage; CBA: challenges the bilingual advantage; JIF: journal impact factor

In order to compare objectively the previously described paper categories, several bibliometric indices were collected. On the one hand, we gathered the accumulated number of citations made to each paper through June 2016, and the number of citations made to each paper per year, which is referred to as ‘citations per year’ (in contrast to ‘accumulated citations’). Thus, ‘citations per year’ includes the raw number of citations made to each paper per year from 2005 to June 2016 (*e*.*g*., number of times that a given paper was cited in 2005). On the other hand, the journal impact factor (JIF) for the year of publication of each study was also considered for the bibliometric analyses.

Finally, the cut-off year was established on 2014 for two reasons. First, the year 2014 has recently been suggested as a cut-off point for a ‘decline effect’ in publication trends with respect to the BA [29]. Secondly, in 2013 was published the most cited study challenging the BA hypothesis [1].

### Data analysis

Three sets of analyses were conducted in order to explore the publication trends in the field of the cognitive advantages associated with bilingualism, and particular interest was given to the comparison between the SBA and the CBA categories. Note that several of the analyses explained in the present section explored temporal changes; however, not all of them used the same temporal windows. On the one hand, ‘time period’ was used to compare two time intervals. As stated earlier, the time intervals (2005 to 2013 and 2014 to 2015) were created not only in accordance with de Bruin and Della Sala [29], but also to acknowledge the year in which the important paper by Paap and Greenberg [1] was published, as it is the most cited CBA paper in the present work. Thus, ‘time period’ was used as a categorical independent factor. On the other hand, we referred to ‘year’ as a within-subjects factor when comparing the dependent variable ‘citations per year’ at three discrete time points (with data ranging from 2014 to June 2016) in a repeated-measures ANOVA.

The first set of analyses explored the distribution of the number of papers in each category (SBA, ABA, NMA, and CBA) by means of the chi-square (χ^2^) statistic. In order to examine whether a differential pattern of publication had taken place, we conducted two χ^2^ tests contrasting paper category and time period: one with the four paper categories, and the other considering only the SBA and CBA categories. Where appropriate, *post hoc* comparisons were made using the methods proposed by Beasley and Schumacker [30]. Additionally, a logistic regression was carried out in order to predict the probability that a paper was published under the CBA category. This regression used as predictors the number of accumulated citations as of June 2016, the year each retrieved study was published, as well as the journal’s JIF for the year of publication.

The second set of analyses explored scores on the different bibliometric indices (*i*.*e*., accumulated citations as of June 2016 and the JIF) as a function of paper category and time period. Mann-Whitney *U*-tests were performed separately for paper categories (SBA and CBA) and time periods (2005 to 2013 and 2014 to 2015) in order to calculate the differences between accumulated citations as of June 2016, and for the JIF. Note that a correlation analysis between the total number of citations and the year of publication showed that papers published earlier had accumulated a larger number of citations, thus providing a reliable picture of the expected trend of citations over time, *r*(139) = -.630, *p* < .0001. Moreover, the correlation between the number of accumulated citations and the JIF for the year of publication showed that papers published in high impact factor journals obtained more citations by June 2016, *r*(139) = .381, *p* < .0001. However, when considering only the papers published in 2014 in the SBA and CBA categories, the latter result was not replicated, *r*(20) = -.171, *p* = .47.

The third set of analyses explored the bibliometric data (citations per year) on the SBA and CBA studies published in 2014, in order to examine the temporal citation trend following both the publication of the paper by Paap and Greenberg [1] and the results of de Bruin and Della Sala [29]. A one-way repeated-measures ANOVA, including paper category (SBA and CBA) as a between-subjects factor and year (2014, 2015, and 2016) as a within-subjects factor, was conducted for the number of citations per year.

SPSS v22 statistical software was used for all statistical analyses. A significance level of *p* < .05 was used for all comparisons, and for all tests of simple effects involving multiple comparisons, a Bonferroni-corrected significance level of *p* < .05 was used.

## Results

**Fig 1:**
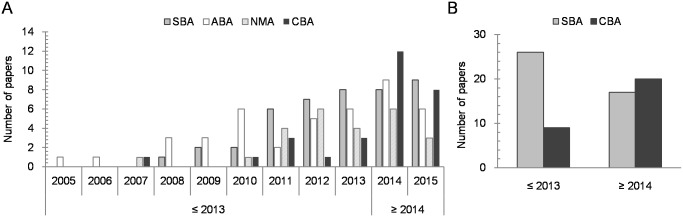
Number of studies published every year in each paper category (A), and number of studies published before and after 2014 in the SBA and CBA categories (B).

The first set of analyses explored the distribution of the number of papers in each category (SBA, ABA, NMA, and CBA; Fig 1A) over time. The χ^2^ test, performed to observe the relationship between the number of papers published in each category (SBA, ABA, NMA, and CBA) as a function of the time period (2005 to 2013 and 2014 to 2015), showed that the paper category was related to time period, with χ^2^(3, *N* = 139) = 9.51, *p* = .023, and *Φ* = .19, revealing, as expected, an overall larger amount of studies published in 2013 and before. Interestingly, *post-hoc* tests revealed that the number of papers published under the CBA category was larger from 2014 onwards, compared to those published between 2005 and 2013 (*p* < .001). No other χ^2^ contrast was significant (*p*s > .16).

When considering only the SBA and CBA categories, the χ^2^ test performed between the two time periods (2005 to 2013 and 2014 to 2015) was also significant, with χ^2^(1, *N* = 72) = 6.01, *p* = .014, and *Φ* = .29. As shown in Fig 1B, the number of CBA studies published in 2014 and later increased (CBA: 31% _2005–2013_ vs. 69% _2014–2015_), while the number of SBA papers published during the same time period decreased (SBA: 60% _2005–2013_ vs. 40% _2014–2015_), compared to those published from 2005 to 2013 in the same categories.

The logistic regression, performed to explore the likelihood that a paper was published under either the CBA or SBA category using bibliometric indices (accumulated citations as of June 2016, the year of publication, and the journal’s JIF for the year that the study was published) as predictors, was statistically significant: χ^2^(3) = 8.33 and *p* = .04. The model explained 12.6% (Nagelkerke *R*^*2*^) of the variance in the publication of paper categories, and correctly classified 65% of cases. Increasing the year of publication was associated with an increased probability (1.47 times) of publishing a paper under the CBA category (*p* = .032). However, neither the accumulated citations, nor the JIF predicted the category under which a paper was published (*p*s > .055).

The second set of analyses examined scores on two different bibliometric indices (*i*.*e*., accumulated citations, JIF). The results showed no statistically significant differences between SBA and CBA categories on the accumulated number of citations as of June 2016 as determined by the Mann-Whitney test: *U* = 586 (*Z* = -0.43), *p* = .666. However, the Mann-Whitney test conducted to compare the accumulated number of citations gathered until June 2016 and time periods (2005 to 2013 and 2014 to 2015), showed that the accumulated number of citations until June 2016 was greater for studies published in 2013 and earlier (*M* = 48.86) than for studies published in 2014 and 2015 (*M* = 24.81), *U* = 215 (*Z* = -4.88), *p* < .0001. Considering the JIF for the year of publication, neither the Mann-Whitney test performed with paper category (SBA and CBA), *U* = 616 (*Z* = -0.08), *p* = .931, nor with time period (2005 to 2013 and 2014 to 2015), *U* = 537.5 (*Z* = -1.24), *p* = .214, revealed significant differences.

**Fig 2:**
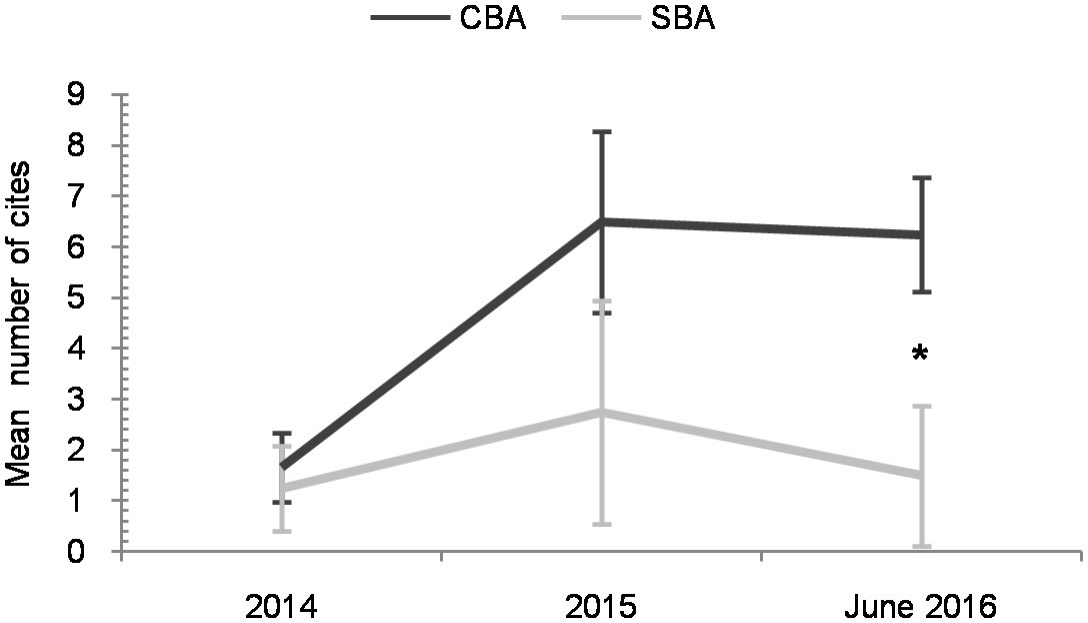
Temporal evolution of mean citations per year of SBA and CBA studies published in 2014. Error bars represent standard errors. Asterisk (* *p* < .05) indicates a significant difference in cites per year between the SBA and CBA categories.

The third set of analyses explored the trend in terms of citations of SBA and CBA studies that were published in 2014. The one-way repeated-measures ANOVA conducted for citations per year included paper category (SBA and CBA) as the between-subjects factor and year (3 time points from 2014 to June 2016) as the within-subjects factor. Results did not reveal a main effect of paper category, *F*(1, 18) = 2.88, *p* = .107, *η*_*p*_^2^ = .363, meaning that the number of citations per year for the CBA category was equivalent to the SBA category. However, a within-subjects effect of year, *F*(2, 36) = 7.03, *p* = .003, *η*_*p*_^2^ = .281, showed that the number of citations per year was greater in both 2015 and June 2016, when compared to 2014 (*p*s < .029), but similar between 2015 and June 2016 (*p* = 1). The interaction between paper category and year was also significant, *F*(2, 36) = 3.3, *p* = .048, *η*_*p*_^2^ = .155. As shown in Fig 2, *post hoc* contrasts showed that citations per year were similar between both paper categories in 2014 and 2015 (*p* = .703, and *p* = .201, respectively), but larger for CBA in June 2016 (*p* = .015).

## Discussion

The present study aimed to present an in-depth analysis regarding several bibliometric indices concerning the literature on bilingualism and cognitive control based on the information retrieved from 139 abstracts. We investigated the pattern of publication of four exclusive paper categories that differed in whether the behavioral information present in the study clearly supported, was ambiguous towards, did not mention, or challenged the BA hypothesis (SBA, ABA, NMA, and CBA, respectively), with a particular interest in the SBA versus CBA comparison. To test the temporal progress of each paper category, we compared bibliometric indices using both two time periods (2005 to 2013 and 2014 to 2015) and three time points (ranging from 2014 to June 2016), taking the year 2014 as a cut-off point. Our findings provide an objective measure that suggests that a change has taken place in the publication trends on the BA.

Data revealed that the pattern of papers published in the CBA category contrasted with that of the other three categories. In this regard, the number of studies published in 2014 and later was smaller than the number published in the previous time period (2005 to 2013) for all paper categories, except for CBA, which an increased number of studies compared to the time period spanning from 2005 to 2013. When considering only the SBA and CBA categories, this change of pattern was still present (see Fig 1B). Although the studies published within the CBA category were rather infrequent in the time period from 2005 to 2013, the publication of CBA studies grew sharply in the 2014 to 2015 time period (see Fig 1A). Regarding the SBA category, the growth rate of SBA publications did not experience a significant variation in the 2014 to 2015 time period (see Fig 1A), showing that results supporting the BA are still reported. Moreover, as predicted by the logistic regression, odds-ratio values indicate that from 2014 onwards it was approximately one and a half times more likely that a new paper would be published in the CBA category. These findings suggest that a change of direction with respect to the BA has taken place, and the study by Paap and Greenberg [1] in 2013 could be considered the turning point for this change, given that it is the most cited CBA paper in the present study. These results may also support the hypothesis presented by de Bruin et al. [20] that a publication bias in favor of positive results supporting the BA hypothesis has influenced this field of publication. Since an increasing amount of studies challenging the BA hypothesis have been published in a two year period, one might argue that results challenging the BA hypothesis were already available but were not likely to be published under the previous literature framework and might have undergone a file drawer effect [31,32].

Other bibliometric aspects analyzed in the present study concern both the quality and impact of CBA papers in the field of bilingualism and cognitive control. The impact and quality of the studies were tested by means of the JIF and two types of citation counts: the number of accumulated citations and the mean number of citations per year. We explored whether the JIF of studies published under the SBA and CBA paper categories or the time period had an influence on publication trends. The impact factor has been found to mediate the quality and relevance of studies in areas such as medicine [33], where studies with better methodological design and analytical methods, among other characteristics, tend to be published in journals with higher impact factors. As the JIF was similar for both publications supporting and challenging the BA, it is reasonable to assume that the overall quality of the papers included was equivalent in both categories and thus, was not a factor that influenced publication tendencies.

There were a similar number of accumulated citations for studies classified as either SBA or CBA, as of June 2016. Therefore a ‘citation bias’, which posits that positive results tend to be more cited [34,35], does not seem to have been confirmed in this area of research. This finding is in line with Callaham et al. [36], who found similar results in medical literature. However, in their study, the JIF was the most relevant quality index. In the present study we found evidence of a relationship between the number of accumulated citations as of June 2016 and the JIF, namely, the higher the JIF of a study, the larger the number of accumulated citations. Nevertheless, neither the JIF nor the accumulated number of citations contributed to the differentiation of the SBA or CBA paper categories. Moreover, studies published in 2013 and before, when mainly SBA papers were published, have accumulated more citations compared to those studies published in 2014 and 2015, as shown by correlational and time period analyses. The present study reports that papers in the CBA category have gained as much influence, in terms of numbers of citations, as those in the SBA category. Regarding the comparison of studies published in 2014, papers in the SBA and CBA paper categories received a similar number of citations per year in 2014 and 2015. Interestingly, CBA papers published in 2014 received a larger number of citations per year in 2016, as compared to the SBA papers published in that same year. Altogether, the results regarding citation number show that there was no difference between SBA and CBA studies in terms of the overall number of accumulated citations. However, CBA papers published in 2014 have accumulated more citations in the last year than SBA papers published in the same year.

Some of the following reasons might explain why there has been a change in the publication trends regarding the BA: the novelty of the results, researchers’ demands for a change of paradigm, or a decline effect in publication trends. As we have shown, results that were indifferent towards or challenged results in terms of a BA were infrequent, but they are not altogether new, as several CBA papers were already published in the 2005–2013 period [19,23]. Thus, novelty may not be the best explanatory factor for the publication shift. Another possible reason is that confidence in the current BA paradigm has been eroded over the last few years. Accordingly, it has been reported that the BA hypothesis does not appear to be robust enough to be adequately replicated [36]. Therefore, researchers might be interested in moving towards a better understanding of the results that call the BA into question, in order to develop further ways to approach this topic. A decline effect could be another plausible explanation for the change in publication trends regarding the BA [29,37]. When a phenomenon is discovered with an initial overestimated effect, it tends to be statistically self-corrected when experiments are repeated, diminishing the early outcome for the sake of a more accurate effect size [38]. This process has been named decline effect, and there are some examples in the psychological literature [39,40]. A key aspect that may contribute to the appearance of decline effects is publication bias [38]. It is not until a phenomenon is settled that it becomes of interest to publish studies against that position, a seemingly leading to a decline effect, at least in terms of effect sizes [40]. In this regard, bibliometric analysis is not a suitable procedure to explore the evolution in effect sizes. Thus, we are not able but to speculate whether the BA literature is undergoing a decline effect. In our point of view, the loss of confidence of the researchers in the current framework, coupled with the effects of a publication bias (which could in turn be a reason for the apparent presence of a decline effect), may be a plausible explanation for the change in the pattern of publication.

Given that the study by Paap and Greenberg [1] has apparently led the path to the publication of null results in this field, one out of three possibilities might occur over the next years. First, challenging results could return to the level of publication they had two years ago, showing that this change on the trends of publication has only been a temporary consequence of a hallmark of the literature. Second, challenging studies continue to overcome those supporting the BA, making the later eventually blur or disappear. Third, hereinafter both challenging and supporting results regarding the BA coexist, being published simultaneously and helping (each from their perspective) to better understand the factors involved in the relationship between cognitive control and bilingualism. In our point of view, the most likely outcome is that CBA studies continue to be published more frequently than SBA articles for some time, as our logistic regression predicts. Probably, some time from now this trend could pause showing a similar publication of SBA, ABA, and CBA studies, depending on the kind of experimental manipulations carried out by the researchers. However, a mid-term follow up would be useful in order to obtain more clear conclusions.

We would like to acknowledge some possible limitations of the current study. First, we based our entire classification criteria only in the information available in the abstracts of each included study. Although similar methods to classify abstracts have been previously adopted (*e*.*g*., [20]), some concerns may rise regarding the reliability of this method. On the one hand, the use of abstracts might be a source of confirmation bias. The information available in the abstracts may be, at least partially, guided by the position of the authors on the BA. To mitigate this potential confound, only the behavioral information provided in the abstract was considered for the classification. In this vein, Pautasso [41] showed that authors in the field of Psychology tend to report a high proportion of null results in their published abstracts. In the case of our study, a considerable amount of the abstracts included in the analyses (see Table 2) reported null, or both null and positive (significant) results, respectively. Therefore, we are confident that our classification criteria captured satisfactorily the current state of the literature. On the other hand, using abstracts as the only source of information might have filtered out a substantial amount of information. However, an abstract is an important reviewed part of a scientific publication, which summarizes the entire article and reflects the most important content of a paper [42], including its most relevant results. While we did not take into account all the results included in the body of the papers, abstracts should include enough representative information in order to classify the studies accurately. Thus, we believe our classification criteria reliably fulfilled the purpose of classifying the abstracts into the appropriated categories. Second, bibliometric analysis does not allow answering some of the questions in the field of the BA (*e*.*g*., whether a decline effect is taking place). The main use of this procedure is to identify and characterize publication trends in terms of scientific impact and production. There are other useful methodologies more suitable to shed light to some of the issues affecting this field (*e*.*g*., the presence of a decline effect), such as meta-analysis [43]. Meta-analysis enables synthesizing the accumulated evidence in a research field, estimating the strength of effects and the variance in the distribution of effect-sizes across the studies analyzed [44]. However, the objective of this study was to provide an overview of the literature regarding the BA, confronting the two more relevant lines of results (*i*.*e*., SBA and CBA). Hence, the decision to use bibliometric analysis was not trivial since it was the most appropriate method to achieve the aim of this study. Despite the previously described limitations, our results provide novel insights on the topic, and describe in a systematic fashion the current state of the literature.

Taken together, the results of the present study seem to confirm the change in the publication patterns of literature on the relationship between bilingualism and cognitive control from a bibliometric perspective. We believe that this redirection of publication tendencies is being led by the need for researchers to satisfactorily incorporate their results into a more robust paradigm, and not just to comply with the BA hypothesis. Publications on enhanced cognitive control attributed to bilinguals have been rather intermittent in recent years. While some authors are able to capture this phenomenon [4,45], others have not been able to do so [46,47] despite applying similar tasks and methods to equivalent samples in terms of age, education, or socioeconomic status. Perhaps the effects of bilingualism on cognitive control have been overestimated in the literature, but this does not indicate that the BA hypothesis is entirely wrong or that the BA does not exist. In fact, although the number of CBA studies published has increased over the past two years, SBA papers are still published at a sustained growth rate. As Bialystok, Abutalebi, Bak, Burke and Kroll [48] stated, “dissenting results are part of the evidence and need to be reconciled with positive findings, not used to overrule them” (p. 57). The existence of studies challenging and supporting the BA should encourage all researchers to be extremely cautious in exploring the factors contributing to the variability of findings regarding the BA hypothesis and also to report all sorts of results arising from well-designed studies with a high degree of control and replicability.

## Supporting information

S1 File (PDF)
