# Supplementary material for: Is bilingualism losing its advantage? A bibliometric approach
Source: PLoS One. 2017 Apr 20;12(4):e0176151. doi: 10.1371/journal.pone.0176151 (PMC5398607; doi:10.1371/journal.pone.0176151)
Supplement: S1 File — (PDF) [file pone.0176151.s001.pdf]

Supporting Information

List of the 139 publications (descending order by year of publication) retrieved from the Scopus database included in our analysis. The search could be replicated by following the query and the inclusion/exclusion criteria of publications reported in the Method section of the manuscript. Each publication is identified by title, abbreviated list of authors, year of publication, journal, journal impact factor for the year of publication (Journal Citation Reports), and accumulated cites in June 2016. See the disposition of the information in the example presented below.

Title				Authors	
Year of publication	*Journal*	Category	% Sup	JIF	Acc. Cites (June 2016)

%Sup: percentage of behavioral information supporting the bilingual advantage; Acc.: accumulated.

Note that paper categories are designated as follows: Supporting the bilingual advantage (SBA) = 1; Ambiguous regarding the bilingual advantage (ABA) = 2; Not mentioning the bilingual advantage (NMA) = 3; Challenging the bilingual advantage (CBA) = 4. Studies classified as NMA have no %Sup.

List of publications

A behavioral and electrophysiological investigation of the effect of bilingualism on lexical ambiguity resolution in young adults				Kousaie et al.	
2015	*Frontiers in Human Neuroscience*	4	0	2,986	0
Audio-visual object search is changed by bilingual experience				Chabal et al.	
2015	*Attention, Perception, and Psychophysics*	1	100	2,168	0
Bilingualism and performance on two widely used developmental neuropsychological test batteries				Karlsson et al.	
2015	*PLoS ONE*	4	0	3,234	0
Feature binding and the processing of global-local shapes in bilingual and monolingual children				Cottini et al.	
2015	*Memory and Cognition*	2	66,7	2,457	2
The impact of late, non-balanced bilingualism on cognitive performance				Vega-Mendoza et al.	
2015	*Cognition*	2	42,8	3,479	8
Attention during visual search: The benefit of bilingualism				Friesen et al.	
2015	*International Journal of Bilingualism*	2	50	0,697	1
Back to Basics: A Bilingual Advantage in Infant Visual Habituation				Singh et al.	
2015	*Child Development*	1	100	4,061	5
Balanced bilingualism and early age of second language acquisition as the underlying mechanisms of a bilingual executive control advantage: Why variations in bilingual experiences matter				Yow & Li	
2015	*Frontiers in Psychology*	3		2,56	7
Bilingual children show an advantage in controlling verbal interference during spoken language comprehension				Filippi et al.	
2015	*Bilingualism*	1	100	2,009	2
Bilingualism and inhibitory control: Possible confounds with the variables "profession" and "level of education"				Rodrigues & Zimmer	
2015	*Calidoscopio*	4	0	0	0

List of publications (continue)

Children's scientific reasoning in the context of bilingualism				Kempert & Hardy	
2015	*International Journal of Bilingualism*	2	50	0,697	0
Cognitive control in bilingual children: Disentangling the effects of second-language proficiency and onset age of acquisition				Struys et al.	
2015	*Swiss Journal of Psychology*	3		0,696	1
Executive control in fluent and lapsed bilinguals				Bogulski et al.	
2015	*Bilingualism*	1	100	2,009	0
Interference control in elderly bilinguals: Appearances can be misleading				Ansaldo et al.	
2015	*Journal of Clinical and Experimental Neuropsychology*	4	0	1,693	3
Linguistic stimulation impact on verbal working memory in the early stages of school education				Pawlicka et al.	
2015	*Acta Neuropsychologica*	2	50	0	0
Musical Training, Bilingualism, and Executive Function: A Closer Look at Task Switching and Dual-Task Performance				Moradzadeh et al.	
2015	*Cognitive Science*	4	0	2,446	0
Proficiency and control in verbal fluency performance across the lifespan for monolinguals and bilinguals				Friesen et al.	
2015	*Language, Cognition and Neuroscience*	1	100	1,47	2
The bilingual advantage in phonetic learning				Antoniou et al.	
2015	*Bilingualism*	1	100	2,009	1
The bilingual advantage in the Stroop task: Simultaneous vs. early bilinguals				Sabourin & Vinerte	
2015	*Bilingualism*	3		2,009	1
The Bilingual Switching Advantage: Sometimes Related to Bilingual Proficiency, Sometimes Not				Tao et al.	
2015	*Journal of the International Neuropsychological Society*	2	33,3	2,963	2
The Development of Prosodic Features and their Contribution to Rhythm Production in Simultaneous Bilinguals				Schmidt & Post	
2015	*Language and Speech*	1	100	1,04	0
The Exposure Advantage: Early Exposure to a Multilingual Environment Promotes Effective Communication				Fan et al.	
2015	*Psychological Science*	1	100	4,94	3
The Joint Effect of Bilingualism and ADHD on Executive Functions				Mor et al.	
2015	*Journal of Attention Disorders*	4	0	3,779	3
The role of executive functions in bilingual children with reading difficulties				Jalali-Moghadam et al.	
2015	*Scandinavian Journal of Psychology*	4	0	1,057	0
Verbal and nonverbal cognitive control in bilinguals and interpreters				Woumans et al.	
2015	*Journal of Experimental Psychology: Learning Memory and Cognition*	1	100	2,862	2
Working memory capacity: Is there a bilingual advantage?				Ratiu & Azuma	
2015	*Journal of Cognitive Psychology*	4	0	1,431	1
Early stage second-language learning improves executive control: Evidence from ERP				Sullivan et al.	
2014	*Brain and Language*	4	0	3,215	8

List of publications (continue)

Bilingualism trains specific brain circuits involved in flexible rule selection and application				Stocco & Prat	
2014	*Brain and Language*	1	100	3,215	3
Are bilingual advantages dependent upon specific tasks or specific bilingual experiences?				Paap et al.	
2014	*Journal of Cognitive Psychology*	4	0	1,431	21
No evidence for reduced Simon cost in elderly bilinguals and bidialectals				Kirk et al.	
2014	*Journal of Cognitive Psychology*	4	0	1,431	9
Electrophysiological explorations of the bilingual advantage: Evidence from a Stroop task				Coderre & Van Heuven	
2014	*PLoS ONE*	1	100	3,234	3
Executive function and bilingualism in young and older adults				Kousaie et al.	
2014	*Frontiers in Behavioral Neuroscience*	4	0	3,27	5
Inhibitory control in bilinguals and musicians: Event related potential (ERP) evidence for experience-specific effects				Moreno et al.	
2014	*PLoS ONE*	4	0	3,234	11
Cognitive advantages and disadvantages in early and late bilinguals				Pelham & Abrams	
2014	*Journal of Experimental Psychology: Learning Memory and Cognition*	1	100	2,862	18
Are bilingual children better at ignoring perceptually misleading information? A novel test				Goldman et al.	
2014	*Developmental Science*	4	0	3,808	1
Better early or late? Examining the influence of age of exposure and language proficiency on executive function in early and late bilinguals				Kalia et al.	
2014	*Journal of Cognitive Psychology*	4	0	1,431	2
Bilingual advantages in executive functioning: Problems in convergent validity, discriminant validity, and the identification of the theoretical constructs				Paap & Sawi	
2014	*Frontiers in Psychology*	4	0	2,56	22
Bilingual children show advantages in nonverbal auditory executive function task				Foy & Mann	
2014	*International Journal of Bilingualism*	2	66,7	0,697	1
Cognitive control in bilinguals: Advantages in Stimulus-Stimulus inhibition				Blumenfeld & Marian	
2014	*Bilingualism*	1	100	2,009	5
Conflict resolution in sentence processing is the same for bilinguals and monolinguals: The role of confirmation bias in testing for bilingual advantages				Paap & Liu	
2014	*Journal of Neurolinguistics*	4	0	1,489	15
Effects of bilingualism and aging on executive function and working memory				Bialystok et al.	
2014	*Psychology and Aging*	1	100	2,646	10
Effects of Bilingualism and Trilingualism in L2 Production: Evidence from Errors and Self-Repairs in Early Balanced Bilingual and Trilingual Adults				Hsu	
2014	*Journal of Psycholinguistic Research*	2	50	0,633	0
Executive function predicts artificial language learning				Kapa & Colombo	
2014	*Journal of Memory and Language*	3		4,237	3

List of publications (continue)

Specificity of the bilingual advantage for memory: Examining cued recall, generalization, and working memory in monolingual, bilingual, and trilingual toddlers				Brito et al.	
2014	*Frontiers in Psychology*	2	25	2,56	1
Inhibitory processes in visual perception: A bilingual advantage				Wimmer & Marx	
2014	*Journal of Experimental Child Psychology*	1	100	2,549	1
Is there a bilingual advantage in the ANT task? Evidence from children				Antón et al.	
2014	*Frontiers in Psychology*	4	0	2,56	33
Learning and processing of nonverbal symbolic information in bilinguals and monolinguals				Blumenfeld & Adams	
2014	*Frontiers in Psychology*	4	0	2,56	0
Maturation of executive functioning skills in early sequential bilingualism				Kalashnikova & Mattock	
2014	*International Journal of Bilingual Education and Bilingualism*	2	33,3	1,027	1
Never too late? An advantage on tests of auditory attention extends to late bilinguals				Bak et al.	
2014	*Frontiers in Psychology*	2	50	2,56	9
Producing bilinguals through immersion education: Development of metalinguistic awareness				Bialystok et al.	
2014	*Applied Psycholinguistics*	2	50	1,512	11
Reassessing the bilingual advantage in theory of mind and its cognitive underpinnings				Nguyen & Astington	
2014	*Bilingualism*	1	100	2,009	4
Second-language fluency predicts native language stroop effects: Evidence from spanish-english bilinguals				Suarez et al.	
2014	*Journal of the International Neuropsychological Society*	3		2,963	1
Specific language impairment in language-minority children from low-income families				Engel De Abreu et al.	
2014	*International Journal of Language and Communication Disorders*	1	100	1,471	1
Flexible memory retrieval in bilingual 6-month-old infants				Brito & Barr	
2014	*Developmental Psychobiology*	2	50	3,307	7
The benefits of being bilingual: Working memory in bilingual Turkish-Dutch children				Blom et al.	
2014	*Journal of Experimental Child Psychology*	2	75	2,549	9
The effect of script similarity on executive control in bilinguals				Coderre & van Heuven	
2014	*Frontiers in Psychology*	2	50	2,56	6
The impact of a third language on executive control processes				Cedden & Simsek	
2014	*International Journal of Bilingualism*	3		0,697	0
The inhibitory advantage in bilingual children revisited: Myth or reality?				Duñabeitia et al.	
2014	*Experimental Psychology*	4	0	2,076	46
The relationship between phonological awareness and executive attention in Chinese-English bilingual children				Yang et al.	
2014	*Cognitive Development*	3		2,172	1

List of publications (continue)

What's Easier: Doing what you want, or being told what to do? Cued versus voluntary language and task switching				Gollan et al.	
2014	*Journal of Experimental Psychology: General*	3		5,929	9
When first language is not first: An functional magnetic resonance imaging investigation of the neural basis of diglossia in Arabic				Nevat et al.	
2014	*European Journal of Neuroscience*	3		3,181	0
The impact of early bilingualism on controlling a language learned late: An ERP study				Martin et al.	
2013	*Frontiers in Psychology*	3		2,56	6
Neurophysiological marker of inhibition distinguishes language groups on a non-linguistic executive function test				Fernandez et al.	
2013	*Brain and Cognition*	4	0	2,477	6
The bilingual L2 advantage in recognition memory				Francis & Strobach	
2013	*Psychonomic Bulletin and Review*	1	100	3,369	0
ERP-pupil size correlations reveal how bilingualism enhances cognitive flexibility				Kuipers & Thierry	
2013	*Cortex*	3		5,128	9
Interference suppression vs. response inhibition: An explanation for the absence of a bilingual advantage in preschoolers' Stroop task performance				Esposito et al.	
2013	*Cognitive Development*	2	50	2,172	5
Where is the bilingual advantage in task-switching?				Hernández et al.	
2013	*Journal of Memory and Language*	2	66,7	4,237	37
A Longitudinal Study of Memory Advantages in Bilinguals				Ljungberg et al.	
2013	*PLoS ONE*	1	80	3,234	10
The effect of dialect experience on Chinese children's Mandarin phonological awareness				Chen et al.	
2013	*Reading and Writing*	4	20	1,634	0
Bilingualism interacts with domain in a working memory task: Evidence from aging				Luo et al.	
2013	*Psychology and Aging*	2	50	2,646	16
Parallel language activation and cognitive control during spoken word recognition in bilinguals				Blumenfeld & Marian	
2013	*Journal of Cognitive Psychology*	3		1,431	18
Tracing the bilingual advantage in cognitive control: The role of flexibility in temporal preparation and category switching				Marzecová et al.	
2013	*Journal of Cognitive Psychology*	1	80	1,431	9
Dual mechanisms of cognitive control in bilinguals and monolinguals				Morales et al.	
2013	*Journal of Cognitive Psychology*	1	100	1,431	16
Attentional control in early and later bilingual children				Kapa & Colombo	
2013	*Cognitive Development*	2	50	2,172	12
Cognitive advantage in children enrolled in a second-language immersion elementary school program for three years				Nicolay & Poncelet	
2013	*Bilingualism*	1	80	2,009	6
The effects of bilingualism on efficiency and lateralization of attentional networks				Marzecová et al.	
2013	*Bilingualism*	1	80	2,009	11

List of publications (continue)

The representation and processing of identical cognates by late bilinguals: RT and ERP effects				Peeters et al.	
2013	*Journal of Memory and Language*	3		4,237	17
The timing and magnitude of Stroop interference and facilitation in monolinguals and bilinguals				Coderre et al.	
2013	*Bilingualism*	2	50	2,009	11
There is no coherent evidence for a bilingual advantage in executive processing				Paap & Greenberg	
2013	*Cognitive Psychology*	4	0	5,064	127
Working memory development in monolingual and bilingual children.				Morales et al.	
2013	*Journal of Experimental Child Psychology*	1	100	2,549	38
Lifelong bilingualism maintains neural efficiency for cognitive control in aging				Gold et al.	
2013	*Journal of Neuroscience*	1	100	6,344	82
Perspective-taking ability in bilingual children: Extending advantages in executive control to spatial reasoning				Greenberg et al.	
2013	*Cognitive Development*	2	50	2,172	16
Executive functions and inhibitory control in multilingual children: Evidence from second-language learners, bilinguals, and trilinguals				Poarch & van Hell	
2012	*Journal of Experimental Child Psychology*	1	100	2,549	19
Influence of bilingualism on memory generalization during infancy				Brito & Barr	
2012	*Developmental Science*	1	100	3,808	18
A bilingual advantage in controlling language interference during sentence comprehension				Filippi et al.	
2012	*Bilingualism*	1	100	2,009	7
Concreteness effects in bilingual and monolingual word learning				Kaushanskaya & Rechtzigel	
2012	*Psychonomic Bulletin and Review*	3		3,369	8
Too much of a good thing: Stronger bilingual inhibition leads to larger lag-2 task repetition costs				Prior	
2012	*Cognition*	2	50	3,479	9
Bilingual recognition memory: Stronger performance but weaker levels-of-processing effects in the less fluent language				Francis & Gutiérrez	
2012	*Memory and Cognition*	3		2,457	1
A bilingual advantage for episodic memory in older adults				Schroeder & Marian	
2012	*Journal of Cognitive Psychology*	1	100	0,756	17
On the time course of exogenous cueing effects in bilinguals: Higher proficiency in a second language is associated with more rapid endogenous disengagement				Mishra et al.	
2012	*Quarterly Journal of Experimental Psychology*	3		2,127	9
Cognitive mechanisms of word learning in bilingual and monolingual adults: The role of phonological memory				Kaushanskaya	
2012	*Bilingualism*	1	100	2,009	5
Subcortical encoding of sound is enhanced in bilinguals and relates to executive function advantages				Krizman et al.	
2012	*Proceedings of the National Academy of Sciences of the United States*	1	100	9,674	62

List of publications (continue)

Where, when and why brain activation differs for bilinguals and monolinguals during picture naming and reading aloud				Jones et al.	
2012	*Cerebral Cortex*	3		8,665	48
The effect of bilingualism on letter and category fluency tasks in primary school children: Advantage or disadvantage?				Kormi-Nouri et al.	
2012	*Bilingualism*	2	50	2,009	3
Conflict monitoring and resolution: Are two languages better than one? Evidence from reaction time and event-related brain potentials				Kousaie & Phillips	
2012	*Brain Research*	4	0	2,843	33
Effects of Marathi-Hindi bilingualism on neuropsychological performance				Kamat et al.	
2012	*Journal of the International Neuropsychological Society*	3		2,963	3
Ageing and bilingualism: Absence of a "bilingual advantage" in Stroop interference in a nonimmigrant sample				Kousaie & Phillips	
2012	*Quarterly Journal of Experimental Psychology*	2	33,3	2,127	37
Effects of early bilingualism on deductive reasoning in elementary school age [Effekte von früher Zweisprachigkeit auf das deduktive Schließen im Grundschulalter]				Kempert & Hardy	
2012	*Zeitschrift fur Entwicklungspsychologie und Padagogische Psychologie*	2	33,3	0,71	0
A Bilingual Advantage in Visual Language Discrimination in Infancy				Sebastián-Gallés et al.	
2012	*Psychological Science*	1	100	4,94	39
Bilingualism Enriches the Poor: Enhanced Cognitive Control in Low-Income Minority Children				Engel de Abreu et al.	
2012	*Psychological Science*	2	50	4,94	47
Emerging bilingualism: Dissociating advantages for metalinguistic awareness and executive control				Bialystok & Barac	
2012	*Cognition*	3		3,479	49
Reasoning about other people's beliefs: Bilinguals have an advantage				Rubio-Fernández & Glucksberg	
2011	*Frontiers in Psychology*	1	100	2,862	16
Inhibition and adjective learning in bilingual and monolingual children				Yoshida et al.	
2011	*Frontiers in Psychology*	1	100	2,56	13
Is there a relationship between language switching and executive functions in bilingualism? Introducing a withingroup analysis approach				Soveri et al.	
2011	*Frontiers in Psychology*	3		2,56	36
The efficiency of attentional networks in early and late bilinguals: The role of age of acquisition				Tao et al.	
2011	*Frontiers in Psychology*	1	100	2,56	51
When the tail counts: The advantage of bilingualism through the ex-Gaussian distribution analysis				Calabria et al.	
2011	*Frontiers in Psychology*	1	100	2,56	4
Cognitive benefits and costs of bilingualism in elementary school students: The case of mathematical word problems				Kempert et al.	
2011	*Journal of Educational Psychology*	4	0	3,518	19

List of publications (continue)

Bilingual advantage in attentional control: Evidence from the forced-attention dichotic listening paradigm				Soveri et al.	
2011	*Bilingualism*	1	100	2,009	19
Early childhood bilingualism leads to advances in executive attention: Dissociating culture and language				Yang et al.	
2011	*Bilingualism*	1	100	2,009	40
Good language-switchers are good task-switchers: Evidence from Spanish-English and Mandarin-English bilinguals				Prior & Gollan	
2011	*Journal of the International Neuropsychological Society*	2	50	2,963	83
Errors on the WCST correlate with language proficiency scores in Spanish-English bilingual children				Vega & Fernandez	
2011	*Archives of Clinical Neuropsychology*	3		1,986	7
Speed of processing, anticipation, inhibition and working memory in bilinguals				Bonifacci et al.	
2011	*Developmental Science*	4	16,7	3,808	14
The effect of bilingualism and age on inhibitory control				Lee Salvatierra & Rosselli	
2011	*International Journal of Bilingualism*	2	50	0,697	25
The effects of bilingualism on toddlers' executive functioning				Poulin-Dubois et al.	
2011	*Journal of Experimental Child Psychology*	4	20	2,549	54
Bilingualism and enhanced attention in early adulthood				Stafford	
2011	*International Journal of Bilingual Education and Bilingualism*	3		1,027	3
Cross-language intrusion errors in aging bilinguals reveal the link between executive control and language selection				Gollan et al.	
2011	*Psychological Science*	3		4,94	33
Bridging language and attention: Brain basis of the impact of bilingualism on cognitive control				Garbin et al.	
2010	*NeuroImage*	1	100	6,357	97
Conflict resolution in sentence processing by bilinguals				Moreno et al.	
2010	*Journal of Neurolinguistics*	4	0	1,742	23
Word mapping and executive functioning in young monolingual and bilingual children				Bialystok et al.	
2010	*Journal of Cognition and Development*	2	50	1,683	47
Cognitive effects of bilingualism: Digging deeper for the contributions of language dominance, linguistic knowledge, socio-economic status and cognitive abilities				Mueller Gathercole et al.	
2010	*International Journal of Bilingual Education and Bilingualism*	2	50	1,027	18
Does bilingualism help memory? Competing effects of verbal ability and executive control				Wodniecka et al.	
2010	*International Journal of Bilingual Education and Bilingualism*	2	75	1,027	18
Advantages of bilinguals over monolinguals in learning a third language				Abu-Rabia & Sanitsky	
2010	*Bilingual Research Journal*	1	100	0	7
Bilingual verbal and nonverbal creative behavior				Kharkhurin	
2010	*International Journal of Bilingualism*	2	50	0,697	18

List of publications (continue)

A bilingual advantage in task switching				Prior & Macwhinney	
2010	*Bilingualism*	2	50	2,009	127
Individual differences in control of language interference in late bilinguals are mainly related to general executive abilities				Festman et al.	
2010	*Behavioral and Brain Functions*	3		1,972	54
Global-Local and Trail-Making Tasks by Monolingual and Bilingual Children: Beyond Inhibition				Bialystok	
2010	*Developmental Psychology*	2	50	4,141	87
On the bilingual advantage in conflict processing: Now you see it, now you don't				Costa et al.	
2009	*Cognition*	2	50	3,479	167
Components of executive control with advantages for bilingual children in two cultures				Bialystok & Viswanathan	
2009	*Cognition*	2	50	3,479	89
Spatial negative priming in bilingualism				Treccani et al.	
2009	*Psychonomic Bulletin and Review*	1	100	3,369	28
Bilingualism and conversational understanding in young children				Siegal et al.	
2009	*Cognition*	1	100	3,479	32
Early bilingualism enhances mechanisms of false-belief reasoning				Kovács	
2009	*Developmental Science*	2	66,7	3,808	67
The source of enhanced cognitive control in bilinguals: Evidence from bimodal bilinguals				Emmorey et al.	
2008	*Psychological Science*	2	50	4,812	80
The effect of childhood bilingualism on episodic and semantic memory tasks: Cognition and Neurosciences				Kormi-Nouri et al.	
2008	*Scandinavian Journal of Psychology*	1	100	1,057	9
Paper: Bilingual experience and executive functioning in young children				Carlson & Meltzoff	
2008	*Developmental Science*	2	33,3	3,808	263
The development of two types of inhibitory control in monolingual and bilingual children				Martin-Rhee & Bialystok	
2008	*Bilingualism*	2	50	2,009	162
What did Simon say? Revisiting the bilingual advantage				Morton & Harper	
2007	*Developmental Science*	4	0	3,808	106
Australian Vietnamese students learning mathematics: High ability bilinguals and their use of their languages				Clarkson	
2007	*Educational Studies in Mathematics*	3		0,579	25
Lexical-semantic organization in bilingual children: Evidence from a repeated word association task				Sheng et al.	
2006	*Journal of Speech, Language, and Hearing Research*	2	66,7	2,07	34
Effect of bilingualism on cognitive control in the Simon task: Evidence from MEG				Bialystok et al.	
2005	*NeuroImage*	2	50	6,357	160
